# Retraction: Computational Characterization of Exogenous MicroRNAs that Can Be Transferred into Human Circulation

**DOI:** 10.1371/journal.pone.0268437

**Published:** 2022-05-09

**Authors:** The PLOS ONE Editors

After this article [1] was published, questions were raised about the methods, data validation, and raw data availability of the study. In light of these issues, *PLOS ONE* reassessed this article with support from a member of the *PLOS ONE* Editorial Board. Based on the outcome of this assessment, the following concerns remain unresolved:

1. The use of a human dataset as a positive training set to predict sequences transferred from other species is insufficient, as it is likely to generate a high number of false positives and negatives.
2. Based on miRNA sequencing analysis data and their alignment with the in silico predictions, the article reports as “transportable milk microRNAs” nine bovine microRNAs identified as enriched in human blood samples collected after cow milk feeding. However, the bovine miRNAs reported as transportable include species that are identical to their human orthologs and so it cannot be discerned whether the enriched miRNAs are derived from cow and/or human. Therefore, the conclusions drawn from these data are not supported.
3. The miRNA sequencing study was not conducted and/or assessed adequately to meet community standards and ensure the reliability of the results. Specifically, it did not include a full evaluation of false and true positives and negatives, a re-analysis of mock/randomized datasets, and/or the validation of microRNAs that have not been described in previous studies.
4. Claims made in the article about “transfer” and “transportability” of miRNAs are not adequately supported. In addition to the issues discussed above, the study does not provide evidence that the identified microRNAs detected have been transported across membranes, as opposed to having been passively released into the bloodstream, e.g. from dead or dying cells.

Following the publication of this article [1], the authors have made the underlying sequencing data for this study available at the NCBI Sequence Read Archive under BioProject ID: PRJNA307561.

In light of these issues, the *PLOS ONE* Editors retract this article due to concerns about the validity of the results, the reliability of the article’s overall conclusions, and the study’s noncompliance with the journal’s 3^rd^ and 4^th^ publication criteria (https://journals.plos.org/plosone/s/criteria-for-publication). We regret that these issues were not identified prior to the article’s publication.

JC and JZ agree with the retraction decision. JS and KC either did not reply directly or could not be reached.
